# Urinary Tract Infections among Bladder Outlet Obstruction Patients in Accra, Ghana: Aetiology, Antibiotic Resistance, and Risk Factors

**DOI:** 10.3390/diseases6030065

**Published:** 2018-07-19

**Authors:** Karikari Asafo-Adjei, James E. Mensah, Appiah-Korang Labi, Nicholas T. K. D. Dayie, Eric S. Donkor

**Affiliations:** 1 Department of Medical Microbiology, School of Biomedical and Allied Health Sciences, University of Ghana, 00233 Accra, Ghana; k.asafoadjei@yahoo.com (K.A.-A.); nicholasdayie@yahoo.com (N.T.K.D.); 2 Department of Surgery, School of Medicine and Dentistry, University of Ghana, 00233 Accra, Ghana; jemensah@hotmail.com; 3 Department of Microbiology, Korle-Bu Teaching Hospital, 00233 Accra, Ghana; guylabi2@gmail.com

**Keywords:** Bladder Outlet Obstruction, urinary tract infection, catheterization, *E. coli*, multi-drug resistant

## Abstract

The aim of this study was to investigate urinary tract infections among patients with Bladder Outlet Obstruction (BOO) at the Korle Bu Teaching Hospital (KBTH) in Accra, Ghana, including the prevalence, risk factors, aetiological agents and their antibiogram. Urine specimens were collected from 188 male patients presenting with BOO and cultured for bacteria. The bacterial isolates were identified using standard microbiological methods and tested against a spectrum of antimicrobial agents using the Kirby Bauer method. Demographic information and the clinical history of study participants were also recorded. The prevalence of urinary tract infection among the BOO patients was 76.6% and the main risk factor identified was catheterization (*p* < 0.0001). A wide range of bacterial organisms was isolated from urine specimens and they were predominantly, Enterobacteriaceae; *Escherichia coli* was the most frequent cause of bacteriuria (33.3%), followed by *Klebsiella* (17.3%). Bacterial isolates were most resistant to Augmentin (97.8%) followed by tetracycline (85.8%), nalidixic acid (82.8%) and ciprofloxacin (75%) while 93.6% were multi-drug resistant. The highest susceptibility was observed with amikacin, which had a resistance prevalence of 4.4% resistance. These findings have important implications in the treatment of urinary tract infections among the BOO patients in Ghana.

## 1. Introduction

The incidence of Bladder Outlet Obstruction (BOO) is high among elderly men, with significant impact on their health and socio-economic wellbeing [1]. An estimated 1.1 billion men are affected by BOO globally with the numbers of affected individuals forecast to increase with time, and the greatest rise is expected in developing regions of the world [1]. The incidence of BOO in Africa is projected to be 21.0% and a prevalence of 13.3% has been reported in Ghana [1,2]. Bladder Outlet Obstruction results in urinary stasis from incomplete voiding. The resultant residual urine serves as a medium for bacteria growth leading to urinary tract infection [3,4,5,6]. Catheterization, which is the commonest mode of easing acute obstructive symptoms in BOO may further introduce extraneous bacteria from the environment or the patient’s own flora [7]. Thus, BOO is a major risk for the development of recurrent urinary tract infection in men [7,8]. The common uropathogens implicated in bacteriuria in BOO patients are *Escherichia coli*, *Pseudomonas* spp., *Staphylococcus aureus*, *Enterococcus* species, *Klebsiella* spp. and *Proteus* spp. [7,9].

In Ghana, antimicrobial treatment of urinary tract infections (UTIs) among BOO patients is mainly empirical due to a relative lack of appropriate laboratory facilities for culture and susceptibility testing of bacteria in several health facilities. Even where laboratory facilities are available, culture and susceptibility tests may not be requested due to cost implications for the patients. Consequently, there is limited data on the uropathogens and their antibiogram among BOO patients in Ghana. Without such surveillance data of antimicrobial susceptibility, empirical treatment of UTIs among BOO patients could be ineffective and expensive. To help address this problem and contribute to the effective management of UTIs among BOO patients, this study was carried out. The aim of the study was to investigate UTIs among patients with BOO at the Korle-Bu Teaching Hospital (KBTH) in Accra, Ghana with the aim of determining the prevalence, risk factors, aetiological agents their antibiogram.

## 2. Materials and Methods

### 2.1. Study Site

The study was conducted at the Korle-Bu Teaching Hospital (KBTH), in Accra. The hospital is presently the leading national referral healthcare facility in Ghana with about a bed capacity of 2000. The hospital has an average daily attendance of 1500 patients and about 250 patients on admission [9]. The facility comprises seventeen clinical and diagnostic departments, which includes the Department of Surgery. The Urology unit is part of the Department of Surgery where subjects for the study were recruited. The unit has an average monthly outpatient attendance of 1000 patients [9].

### 2.2. Study Design, Subject Recruitment, and Data Collection

This was a cross-sectional study involving 188 male BOO patients at KBTH conducted from January to June 2017. The participants were recruited from patients with BOO who came for a change of the in-situ catheter and BOO patients who came for urine flow/urodynamics studies. Clinically and/or sonographically diagnosed males with BOO of age 40 years and above, who consent to be part of the study were enrolled. Patients on antibiotic treatment within two weeks prior to sample collection were excluded. Demographic data and clinical history of the study subjects were extracted from their clinical records with the consent of the participants and clinical staff. The information included age, occupation, level of education, previous urinary tract infection, history of catheterization, co-morbidities and antibiotic usage.

### 2.3. Laboratory Analysis

A wet mount of uncentrifuged urine was prepared and observed under a microscope for pus cells, red blood cells, yeast cells, and white blood cells (WBC) cast. Urine samples were further analyzed to detect the presence of nitrite, leukocytes, blood and protein and other biochemical parameters urine dipstick test strips within two hours of sample collection. The urine specimens were quickly cultured on Cysteine Lactose Electrolyte Deficient (Oxoid Ltd., Basingstoke, UK) media and incubated at 37 °C for 18–24 h and assessed for significant bacteriuria [10] at the Department of Medical Microbiology, School of Biomedical and Allied Health Sciences. Following incubation, colonies of bacterials were counted and counts of ≥10^5^ (cfu/mL) were regarded as significant bacteriuria [11]. Isolated bacteria were identified based on their colonial morphology, Gram’s staining reactions and biochemical tests including oxidase test, triple sugar iron (TSI) fermentation tests, indole test, citrate utilization test, urea utilization test, catalase test, coagulase test and motility. After analysis of the urine specimens, the urinary tract infection in the study participants was determined by the significant bacteriuria with the presence of pyuria [12].

The antimicrobial susceptibility testing was performed on urinary isolates from specimens with significant bacteriuria using the Kirby Bauer disc diffusion method and the Clinical Laboratory Standard Institute guidelines [13]. The antibiotics disc and their concentrations used for the study were Augmentin 30 µg, piperacillin 20 µg, ceftriaxone 30 µg, ceftazidime 20 µg, nalidixic acid 30 µg, ciprofloxacin 5 µg, levofloxacin 5 µg, norfloxacin 20 µg, gentamicin 10 µg, amikacin 30 µg, nitrofurantoin 300 µg and tetracycline 30 µg (Biomark Laboratories, Pune, India). Standardized inoculums were prepared in sterile saline to turbidity comparable to 0.5 McFarland standard solution of barium sulfate. Muller-Hinton agar plates were swabbed with the standardized inoculums and antimicrobial discs aseptically placed on it after drying the plate for 3–5 min. The plates were then incubated at 37 °C under aerobic conditions for 16–18 h as per 2016 Clinical Laboratory Standard Institute guidelines [13,14]. The zone of inhibition for each antimicrobial was measured with a pair of calipers in millimeter and interpreted as sensitive, intermediate and resistant according to CLSI 2016 breakpoints [13]. To determine the percentage of resistant strains to each antibiotic, resistant and intermediate isolates were classified together as resistant in this study [15]. *Escherichia coli* (ATCC 25922) was used to quality control the antibiotic discs.

### 2.4. Data Analysis

Data from the study was entered in Microsoft Excel 2010 and analyzed in STATA 12.0. A descriptive analysis including the computation of arithmetic means, frequencies, and percentages was done with the study variables. Univariable associations were performed between the urinary tract infection and the other study variables. Analysis of variance was used for numeric variables, whereas the chi-square test was used for categorical variables. Variables significantly associated with urinary tract infection in the invariable analysis were used as the independent variables in a logistic regression analysis to identify determinants of the urinary tract infection; *p*-values < 0.05 were regarded as significant.

### 2.5. Ethics Statement

Ethical approval for the study was sought from the Ethical and Protocol Review Committee of the College of Health Sciences, University of Ghana and the Institutional Review Board of Korle-Bu Teaching Hospital. Urine samples, as well as demographic information, were obtained from study participants following their consent to partake in the study.

## 3. Results

### 3.1. Demographic and Clinical Features of the Study Participants

A total of one hundred and eighty-eight (188) males with BOO participated in the study and their demographic features are summarized in Table 1. The age range of study participants was 40 to 94 years with an average age of 69.1 ± 10.45 years. The most common educational level attained by study participants was primary (36.7%) followed by senior secondary school (30.3%). In terms of occupation, most of the study participants (61.7%) were pensioners followed distantly by artisans (21; 11.2%) and sales workers (13; 6.9%).

The most frequent cause of BOO was benign prostatic hyperplasia, which affected 139 (73.9%) of the 188 study participants, followed by urethral stricture (13.3%) and prostate cancer (12.8%). The most prevalent comorbidity among BOO patients was hypertension (36.2%) followed by diabetes (8.0%) and strokes (4.3%). Furthermore, 29.3% of the BOO patients had a urinary tract infection within one year of the study. The most commonly prescribed antibiotics for the BOO patients were ciprofloxacin (28.7%), nitrofurantoin (6.4%) and gentamicin (3.2%). One hundred and fifty-seven (83.5%) study participants had an indwelling urinary catheter with 77 (41.0%) of them catheterized within one year, 25 (13.3%) above one to two years, and 21 (11.2%) above five years. One hundred and forty (89.2%) of the catheterized participants changed their catheters every 3 weeks while the remaining 17 (10.8%) changed their catheters beyond every three weeks.

### 3.2. Bacteriuria and Associated Risk Factors

Overall, 144 of the 188 BOO patients had UTIs, which translates to a prevalence of 76.6% (95% CI: 70.1 to 82.1). The univariable analysis showed that none of the demographic features of the study participants including age, occupation, and education were associated with significant bacteriuria (*p* > 0.05). Two clinical features of the study participants including catheterization and diabetes were significantly associated with urinary tract infection in the invariable analysis (*p* < 0.05). In the logistic regression analysis, catheterization emerged as a predictor of urinary tract infection (*p* < 0.001).

### 3.3. Uropathogens and Antibiotic Susceptibility

A wide range of bacterial organisms was isolated from urine specimens of the BOO patients that had significant bacteriuria and were predominantly Enterobacteriaceae (Table 2). *E. coli* was the most frequent cause of bacteriuria (33.3%), followed by *Klebsiella* (17.3%), which comprised 9 cases of *K. pneumoniae* and 18 cases of other *Klebsiella* species. Apart from Enterobacteriaceae, *Staphylococcus* and *Pseudomonas* were also isolated from urine specimens of the BOO patients but these organisms were isolated in small numbers. There were 15 (9.6%) bacteriuria cases due to *Pseudomonas*, which comprised of 6 cases of *P. aeruginosa* and 9 cases of other *Pseudomonas* spp. and 7 (4.5%) cases of *Staphylococcus* (4.5%) which comprised of 1 isolate of *S. aureus* and 6 isolates of coagulase-negative *Staphylococcus*.

The antibiogram of the different bacterial organisms isolated from the urine specimens is reported in Table 3. Bacterial isolates were mostly resistant to Augmentin (97.8%) followed by tetracycline (85.9%), nalidixic acid (82.8%) and ciprofloxacin (75%). However, the highest susceptibility was observed with amikacin (6.4%). The mean resistance observed among bacterial isolates was *E. coli* (62.3%), *Klebsiella* spp. (58.9%), *Citrobacter* spp. (60.6%), *Enterobacter* spp. (55.6%), *Pseudomonas* spp. (39.1%) and *Proteus* spp. (56.1%). An overall mean resistance of 58.5% was observed among isolates against the twelve antibiotics used in the study. All *E. coli* were resistant to Augmentin and a 92.3% resistance was observed with ciprofloxacin, nalidixic acid, and tetracycline. Most *E. coli* were, however, susceptible to amikacin and nitrofurantoin with 3.8% and 17.3%, respectively. The highest resistance to amikacin was observed among the *Pseudomonas species* (22.2%). Prevalence of multi-drug resistance and extended-spectrum beta-lactamase (ESBL) among the bacteria isolates are reported in Table 3. An overall multi-drug resistance prevalence of 93.6% was observed while 46% of the isolates screened for ESBL were positive. In *E. coli*, the commonest isolate, the prevalence of multidrug resistance and ESBL were 98.1% and 44.2%, respectively.

## 4. Discussion

The focus of this study was to provide data on antibiotic resistance of uropathogens associated with BOO patients with the goal of improving treatment outcomes of urinary tract infections in the patients. The mean age (69.1 years) of the participants in this study is similar to that of a previous study on urinary tract infection among BOO patients in Kumasi, Ghana in which the mean age reported was 62.0 years [8,16]. The most frequent cause of BOO in this study were benign prostatic hyperplasia (73.9%) and urethral stricture (13.3%), which concurs with the previous study in Kumasi, which reported 76.7% prostatic hyperplasia prevalence and 22.3% urethral stricture [8,16]. The prevalence of urinary tract infection among BOO patients in this study (76.6%) is much higher than that reported in Kumasi (40.2%) by Gyasi-Sarpong et al., 2014 [8]. Compared to the study by Gyasi-Sarpong et al., (2014), most of the participants (83.5%) in our study had chronic indwelling urinary catheterization and this may have accounted for the relatively higher prevalence observed. This is confirmed by the strong statistical association between catheterization and urinary tract infection observed in our study. Several studies have documented significant association between urinary tract infection and catheterization [6,17,18]. In Nigeria, a study reported a 88.5% prevalence of urinary tract infection among catheterized individuals [18], which is consistent with the 89.2% (140/157) prevalence among catheterized participants in this study. The process of catheterization leads to the introduction of endogenous or exogenous bacteria into the urinary tract resulting in bacteriuria [19,20]. Additionally, the formation of bacterial biofilms on the surfaces of catheters results in chronic bacteriuria, which are difficult to treat [21]. 

In line with several other studies, *E. coli* was the commonest cause of urinary tract infection [18,22,23]. Alongside *E. coli*, other Enterobacteriaceae particularly, *Klebsiella*, *Enterobacter*, and *Citrobacter* accounted for about 70% of the bacterial etiology of urinary tract infection in BOO patients. This pattern of urinary tract infection etiology is similar to that previously reported among BOO patients in Ghana and could facilitate the development of empirical antibiotic treatment of bacteriuria among these patients. The high prevalence of resistance to Augmentin, tetracycline, and fluoroquinolones observed in this study could be due to the high rate of use of these drugs in Ghana as they have been in the market for a long period of time. This concurs with the general trend of escalating antibiotic resistance in Ghana [24,25,26,27,28,29,30,31]. Interestingly, Ciprofloxacin, which is the most commonly prescribed antibiotic for the study participants, showed a high prevalence of resistance (50–92.3%) and is therefore unsuitable for empirical treatment of UTIs in the BOO patients. Unfortunately, *E. coli*, the commonest uropathogen in this study, showed the highest prevalence of resistance to Ciprofloxacin (92.3%) and this suggests a high probability of treatment failures of UTIs among the BOO patients. The prevalence of Ciprofloxacin resistance in this study concurs with a nationwide antibiotic resistance study in Ghana in 2015 that reported a Ciprofloxacin resistance of >50% for *E. coli* and other Enterobacteriacae [30]. Ciprofloxacin is one of the prescribed antibiotics in Ghana due to the appreciable levels of susceptibility of bacterial pathogens such as *E. coli* and *Salmonella typhi* to the antibiotic in the past [29,32]. However, recent epidemiological evidence including this study calls for a review of the use of Ciprofloxacin in the empirical treatment in Ghana. In our dataset, *E. coli* resistance to Amikacin was very low (<4%) which indicates that this antibiotic is relatively suitable to be used in the empirical treatment of UTIs among BOO patients in Ghana. This agrees with the findings of other studies where amikacin was highly effective against ESBL-producing and quinolone-resistant uropathogenic *E. coli* [26,27]. However, the major issue of amikacin and other aminoglycosides use relates to their toxicity, i.e., ototoxicity and nephrotoxicity [33,34]. Thus, aminoglycosides are contraindicated in patients with renal impairment [34]. Urinary tract infections in patients with renal failure are usually recurrent and more difficult to eradicate. In such cases, combination therapy would perhaps be ideal. A study showed that the combination of fosfomycin and amikacin or ceftazidime was an effective therapeutic and preventive strategy in children with vesicoureteral reflux and recurrent relapsing urinary tract infection [35]. Antibiotic treatment outcomes are adversely affected by the presence of risk factors such as catheterization and, therefore, urinary catheters should be replaced before initiating antimicrobial therapy [19].

## 5. Conclusions

Urinary tract infection is a common complication among BOO patients at KBTH and the main associated risk factor is catheterization. *Escherichia coli* is the main cause of UTIs among these patients and the infections could be treated with amikacin. Further studies and monitoring for potential toxicity relative to local epidemiology is important for the successful use of this therapy. Ciprofloxacin, which is the most commonly prescribed antibiotic for the empirical treatment of UTIs among BOO patients at the study hospital currently, is less effective.

## Figures and Tables

**Table 1:** The demographic and clinical features of the study participants.

Feature	*n*	%
Age (mean = 69.1 ± 10.5 years)		
Education		
Primary	69	36.7
Junior Secondary School	3	1.6
Senior Secondary School	55	29.3
Tertiary	30	16
None	31	16.4
Occupation		
Pensioner	116	61.7
Unemployed	2	1.1
Artisan	21	11.1
Sales worker	13	6.9
Agricultural worker	9	4.8
Driver	9	4.8
Security worker	6	3.2
Civil servants	6	3.2
∗ Others	6	3.2
Cause of the BOO		
Benign prostatic hyperplasia	139	73.9
Prostate cancer	24	12.8
Urethral strictures	25	13.3
Comorbidities		
Hypertension	68	36.2
Diabetes	15	8
Stroke	8	4.3
‡ Others	3	1.6
Chronic urinary catheterization	157	89.2
Duration of catheterization (Range 3 weeks–30 years)		
≤1 year	77	41
>1–2 years	25	13.3
>2–3 years	12	6.4
>3–4 years	13	6.9
>4–5 years	9	4.8
>5 years	21	11.2
Sign of bacteriuria		
Pyuria	158	84
Haematuria	96	51.1
Nitrite	42	22.3
Urinary tract infection ≤1 year	55	29.3
Antibiotic usage ≤1 year	70	37.2

BOO = bladder outlet obstruction, “*n*” = number of study subjects; * Others = engineers, auto mechanic and barber; ‡ Others = asthma and hernia.

**Table 2:** The bacteria isolated from urine samples of the bladder outlet obstruction patients.

Bacterial Isolates	*n*	%
*Escherichia coli*	52	33.3
*Klebsiella* species	18	11.5
*Enterobacter* species	15	9.6
*Klebsiella pneumoniae*	9	5.8
*Proteus mirabilis*	9	5.8
*Pseudomonas* species	9	5.8
*Citrobacter* species	8	5.1
*Citrobacter koseri*	6	3.9
Coagulase negative *Staphylococcus*	6	3.9
*Pseudomonas aeruginosa*	6	3.9
*Providencia rettgeri*	5	3.2
*Klebsiella oxytoca*	4	2.6
*Proteus vulgaris*	2	1.3
*Serratia marcescens*	2	1.3
*Citrobacter freundi*	1	0.6
*Providencia* species	1	0.6
*Salmonella* species	1	0.6
*Salmonella typhi*	1	0.6
*Staphylococcus aureus*	1	0.6

“*n*” = number of urine specimens.

**Table 3:** The prevalence of antimicrobial resistance among bacterial isolates.

	Resistance of the Isolates to Antibiotics (%)									
Antibiotic	*EC* (*n* = 52)	*KL* (*n* = 31)	*CS* (*n* = 15)	*ES* (*n* = 15)	*PS* (*n* = 15)	*PR* (*n* = 11)	*PV* (*n* = 6)	*ST* (*n* = 7)	*SM* (*n* = 2)	*SS* (*n* = 2)
AUG	52 (100)	29 (93.6)	15 (100)	15 (100)	NA	10 (90.9)	6 (100)	NA	2 (100)	2 (100)
PIP	23 (44.2)	16 (51.6)	4 (26.7)	6 (40)	5 (33.3)	2 (18.2)	3 (50)	NA	0 (0)	1 (50)
CEF	25 (48.1)	15 (48.4)	7 (46.7)	7 (46.7)	NA	5 (45.5)	2 (33.3)	NA	0 (0)	2 (100)
CFT	25 (48.1)	18 (58.1)	6 (40)	5 (33.3)	8 (53.3)	4 (36.4)	2 (33.3)	NA	0 (0)	2 (100)
NAL	48 (92.3)	24 (77.4)	15 (100)	11 (73.3)	NA	9 (81.8)	4 (66.7)	NA	2 (100)	2 (100)
CIP	48 (92.3)	23 (74.2)	12 (80)	11 (73.3)	6 (40)	7 (63.6)	3 (50)	5 (71.4)	1 (50)	1 (50)
LEV	45 (86.5)	20 (64.5)	10 (66.7)	9 (60)	6 (40)	6 (54.6)	3 (50)	5 (71.4)	1 (50)	1 (50)
NOR	44 (84.6)	20 (64.5)	10 (66.7)	8 (53.3)	7 (46.7)	6 (45.6)	4 (66.7)	5 (71.4)	1 (50)	2 (100)
GEN	20 (38.5)	11 (35.5)	4 (26.7)	7 (46.7)	6 (40)	6 (54.6)	1 (16.7)	4 (57.1)	1 (50)	0 (0)
AMK	2 (3.8)	1( 3.2)	0 (0)	0 (0)	3 (20)	0 (0)	1 (16.7)	1 (12.3)	1 (50)	1 (50)
NIT	9 (17.3)	20 (64.5)	12 (80)	8 (53.3)	NA	9 (81.8)	5 (83.3)	5 (71.4)	2 (100)	2 (100)
TET	48 (92.3)	22 (71)	14 (93.3)	13 (86.7)	NA	10 (90.9)	5 (83.3)	6 (85.7)	1 (50)	2 (100)
RM (%)	62.3	58.9	60.6	55.6	39.1	56.1	54.2	63.3	50	75

NA = not applicable; EC = E. coli; KL = Klebsiella spp.; CS = Citrobacter spp.; ES = Enterobacter spp.; PS = Pseudomonas spp.; PR = Proteus spp.; PV = Providencia species; ST = Staphylococcus spp.; SM = Serratia marcescens; SS = Salmonella species; AMK = amikacin; AUG = Augmentin; CEF = ceftriaxone; CFT = ceftazidime; CIP = ciprofloxacin; GEN = gentamicin; LEV = levofloxacin; NAL = nalidixic acid, NIT = nitrofurantoin; NOR = norfloxacin; PIP = piperacillin; TET = tetracycline; RM = mean resistance.
